# Choice Wheat Product

**Published:** 1875-10

**Authors:** 


### A CHOICE WHEAT PRODUCT.

The Health Food Company have added a new article of choice food to their list of good things. They have been seeking the most perfect whole wheat flour to be found, and believe they have it at last.

It is merely choice, sound, matured wheat, very thoroughly cleansed,—polished, in fact, and reduced to a fine powder. It has no trace of dirt, or seeds of noxious weeds, or other extraneous matter, in a ton of it. It is wheat, whole wheat, and nothing but wheat, and wheat at its best.

The processes employed are those of the Cold-Air Attrition Mills, 300 South Clark Street, Chicago, of which mills W. Warren, Esq., is manager.

The wheat is the best which can be obtained at any price in that great grain centre, Chicago; and the pledge given by all concerned, is, that the standard of superior excellence shall be conscientiously adhered to.

The first process—that of cleansing the grain—is found to be one of great importance, with reference to the flavor and digestibility of wheat products. Great care is therefore taken at this preliminary stage. All the ordinary smut and scouring machinery, and air-blasts, are first used in the preparation of this wheat, and it is then submitted to new and more efficient processes, in order to secure the removal not only of all unclean and deleterious matters, but also all absolutely inert and useless elements. The grains of wheat, when ready for the flouring process, evince a notable loss of the woody and silicious husk which envelope them in their commercial state. All that remains after the several processes has its useful and appropriate place in the economy of life.

We come next to the flouring process; and this we find to be unique, efficient, and wonderfully interesting. There is no grinding between millstones—there is no grinding process of any kind made use of; what is called "attrition" is used, but this term expresses the operation but poorly. It is really explosion—the instantaneous bursting of the grain by the force of the terrific cold-air currents. It is as if the grains were seized by opposing blasts, and dashed to atoms against one another by the force exerted.

That there is a great advantage in this means of pulverizing, over the ordinary millstone plan, must be evident. The microscope—which is the court of last resort in all scientific researches—reveals a remarkable fact here. It shows that every particle of mill-flour is flat, while every atom of this attrition flour is spherical. The latter is light and airy in appearance, while that which has passed between millstones looks compact, and heavy, and solid, under the strong magnifier. The one would be chosen—if the vision alone were consulted—as a delicate and readily soluble food; the other would be rejected as heavy and indigestible.

But there are other advantages connected with this new process, and they are of vast importance. Millstones by which wheat is ground, become so hot from the immense friction involved, that you can not bear your hand on them. Flour, as it is discharged from the stones, is found to be very hot. This fact has long been known to greatly deteriorate the product. Hundreds of patents have been issued for methods by which this heating effect can be avoided. The stones are run slowly or far apart, and repeated grindings are had; in short, so grievous is the injury done to the flour by the elevation of temperature consequent upon grinding, that every effort has been made to overcome the tendency to it. Still, the heat will be generated if friction occurs, and friction must occur in all grinding-mills. The harm done by friction is vast. To heat a mass of flour, and to allow it to remain in a compact mass, is to plant the seeds of destruction in its bosom. Decomposition of vegetable substances is always attended with heat, and any grain or grain-product which has reached the heated stage, has taken a long stride toward death. Moreover, it is reasonable to believe that the rude tearing asunder of the delicate structure of the grain cannot be advantageous; and the rapid wearing of the millstones proves conclusively that no little injurious extraneous matter is mingled with the flour through the destruction of the surfaces of the stones. French chemists have detected essential principles in wheat which ordinary flour lacks, and they declare that the cereals are greatly lessened in value as brain and muscle sustaining food, by the grinding process.

If nature has properly arranged the proportions of the several ingredients entering into the structure of wheat, so as to form the best food for sustaining vigorous human life, then it is obvious that we can not change those relative proportions without injury. We can not rob the wheat of one element without lessening its vital power. If we weaken the force of one constituent we impoverish our food to that extent. If we add—by our processes of preparation or of cooking—injurious substances, to that extent we deteriorate our food. Now one or all of these bad effects are induced in most wheat products. In superfine flour we rob the grain of its best elements—the nitrogen and the phosphates. We absolutely steal from ourselves the qualities which give us brain-power, nerve-force, and strength to labor. By our system of grinding our grain, we lessen its vitality and its life-sustaining power. We add to it gritty and limey particles—worn from the millstones—to irritate our stomachs. To crown all, we weaken and often destroy whatever of excellence remains, by our cooking-processes.

The Cold Air Attrition process, and its accompanying methods of cleansing, add nothing, take away only foreign and inert matter, and change nothing. The vast volumes of rushing air at low temperature keeps down all heat. As a result the properties in the wheat are found in the flour, atom for atom, the nitrogenous, the phosphatic, and the carbonaceous, unchanged and uncontaminated. If good clean wheat is a good food, then this flour must be excellent. The cold air brought in forcible contact many times with each separate particle of the flour, perfects and ærates it and adds marvellously to its keeping qualities. Besides, so much of the outer coat as remains after the cleansing and polishing processes, is reduced to a powder of equal fineness with the more pulverulent starchy parts. There are no harsh and husky particles to irritate stomachs having sensitive lining membranes. The most delicate child may eat food made from this flour with entire comfort; and all children acquire a real fondness for bread and mush and cake thus made.

Its economy is important. It is used largely in the army, where care is taken to secure nutriment in its most compact form. A letter lately received from Surgeon-General Barnes, awards it the highest praise. It is found to make more bread, as well as better bread, than any flour ever before known. Even careless bakers make as high as 316 lbs. of bread from 196 lbs. of the Cold-Air Attrition flour. In the family, where care is used, a larger yield is secured.

This product is rich in gluten, and expands greatly in mixing. It demands different treatment from the common starchy flour. It must be stirred with much water to the consistency of a thin batter, and not kneaded at all. It is not a laborious task to convert it into the most appetizing and delicious bread.

Another article put up by the same Company is the Pearled Wheat. This is the pure, polished grain, denuded of its husk. It is the most perfect substitute for "cracked wheat," "crushed wheat," or "wheaten grits," which we have ever seen. Properly boiled, it makes a delightful food. It is kept by the HEALTH FOOD COMPANY, 137 Eighth Street, who also have the best form of jacketed kettle, or double boiler, in which to prepare it.

